# Health by Act of Parliament

**Published:** 1895-03-16

**Authors:** 


### The Hospital

An Institutional Journal of

The Medical Sciences and Hospital Administration, with

AN EXTRA NURSING SUPPLEMENT.

Vol. XVII.—No. 442.] [March 16, 1895.

### Health by Act of Parliament.

The community, as a community, is under the constant necessity of protecting itself against the individual. The individual, in the last appeal, will be found to be a fighter for his own hand. If he is called upon to choose between his own interests and those of the community he will certainly prefer his own, notwithstanding any ridiculousness which may appear in such a choice when the two sets of interests are placed side by side for comparison. That being so, the community, if it has attained to any considerable degree of mental force and capacity as a community, will take care to watch with all necessary keenness its communal interests, exactly as the individual takes care to set a sufficient guard over his own. A reference to general principles of this order is commonly necessary if we are to form anything like adequate judgments on important legislative changes which may be proposed in the interests of communities as against those of limited classes or separate individuals. We would ask readers to bear in mind these general principles in considering the somewhat novel but highly important subject of sanitary registration.

Sanitary registration is an idea with which all sanitarians, and most persons who have travelled any considerable distance from their own homes, are quite familiar. Not until recently has any vigorous attempt been made to render such registration compulsory by statute. A Bill has now reached the stage of being printed which will shortly be brought before Parliament, and all practical politicians of every party should feel themselves bound to study the question in order that legislation on the subject may be both practical and reasonable. The Act is intended to provide for the "sanitary registration of dwelling-houses, schools, colleges, hospitals, asylums, wash-houses, factories, hotels, lodging-houses, and other buildings within the United Kingdom." The Bill, in brief, recognises that there are certain dangers to health and life which spring from the single fact that we are mainly an indoor population. All of us sleep indoors; most of us work indoors. Let us, therefore, see to it that so far as science and legislation can help us we will have nothing indoors with us which may endanger health and life.

In matters of this kind most of us are utilitarian and practical. The questions we naturally ask ourselves are these: Will the registration of all sorts of places in which we lodge or live permanently be of real service to the general health of the public? And will the service be of such kind and degree as to compensate us for the inconvenience such registration will inflict upon individuals and limited classes, and also for the cost it will impose upon ourselves? For we must not close our eyes to the facts that sanitary registration will press somewhat hardly upon certain individuals and limited classes, and, moreover, cannot but entail considerable cost.

In order that we may judge reasonably of the necessity and worth of registration, we must first satisfy ourselves on the question of what registration can and will do for us. It will secure for us a competent periodical inspection of the sanitary condition of dwelling-houses and all other places wherein men and women lodge or live permanently, and it will compel owners of property to act with due regard for the health and interests of those who may rent their property. Now, on the general question, as to whether owners of house property should be compelled to give a kind of practical warranty of the healthiness of their property, all that is necessary is to bear in mind that all other classes who have anything to sell to the public are compelled to act upon principles of this order. The Food Adulteration Acts are merely Acts to compel sellers, in the interests of buyers, to sell that which they profess to sell, and nothing else. Similarly, the Weights and Measures Acts are designed to compel vendors, in the interests of purchasers, to give just weight and nothing else. Whatever may be said in favour of Food Adulteration Acts, Weights and Measures Acts, and the like, may be said with precisely the same degree of reasonableness and justice in favour of compelling property owners, letters of lodgings, *et hoc genus omne*, to provide the wholesome and healthy houses which they profess to provide, and nothing else.

But supposing all this to be admitted, there still remains the further practical question: Is registration of dwellings and other like places the most simple and effectual method of attaining the end in view? Let us answer this question by asking another: What other method than that of registration would be at once so simple, easy, and effectual? So far as we know this is the only method which can by any possibility meet the necessities of the case. Registration, the registration of all kinds of buildings wherein men and women in our densely-populated country live and work, seems to us to be a distinct movement forward in the direction of a higher civilisation and the better protection of human life. It is for all of us to reason out the subject to legitimate conclusions, and then to insist that that which is so manifestly for the good of the community shall be made a permanent addition to the statute book with as little delay as possible.

